# Top-down regulation of motivated behaviors via lateral septum sub-circuits

**DOI:** 10.1038/s41380-022-01599-3

**Published:** 2022-05-18

**Authors:** Antoine Besnard, Felix Leroy

**Affiliations:** 1 IGF, Univ Montpellier, CNRS, Inserm, Montpellier, France; 2 Instituto de Neurociencias CSIC-UMH, San Juan de Alicante, Spain

**Keywords:** Neuroscience, Molecular biology

## Abstract

How does cognition regulate innate behaviors? While the cognitive functions of the cortex have been extensively studied, we know much less about how cognition can regulate innate motivated behaviors to fulfill physiological, safety and social needs. Selection of appropriate motivated behaviors depends on external stimuli and past experiences that helps to scale priorities. With its abundant inputs from neocortical and allocortical regions, the lateral septum (LS) is ideally positioned to integrate perception and experience signals in order to regulate the activity of hypothalamic and midbrain nuclei that control motivated behaviors. In addition, LS receives numerous subcortical modulatory inputs, which represent the animal internal states and also participate in this regulation. In this perspective, we argue that LS sub-circuits regulate distinct motivated behaviors by integrating neural activity from neocortical, allocortical and neuromodulatory inputs. In addition, we propose that lateral inhibition between LS sub-circuits may allow the emergence of functional units that orchestrates competing motivated behaviors.

## Revisiting LS anatomy and connectivity

**Fig. 1:**
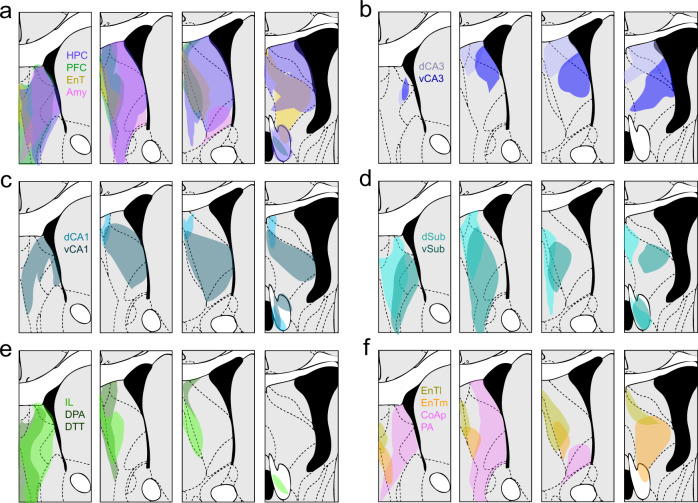
Organization of LS cortical excitatory inputs. **a** Summary of main neocortical (cortical regions with six layers) and allocortical (cortical regions with three layers) afferences to LS. **b–f** Organization of hippocampal (**b**–**d**), frontal (**e**) and temporal (**f**) afferences to LS. Data were obtained from the Mouse Brain Connectivity Atlas (connectivity.brain-map.org) mapped at the level of four LS coronal planes (+1.1, +0.62, +0.38 and −0.1 mm from Bregma). Amy: amygdala, CoAp: posterior cortical amygdala, DBN: diagonal band nucleus, DPA: dorsal peduncular area, DTT: dorsal tenia tecta, EnT: entorhinal cortex, HPC: hippocampus, IL: infralimbic cortex, PA: posterior amygdala, PFC: prefrontal cortex, Sub: subiculum.

**Fig. 2:**
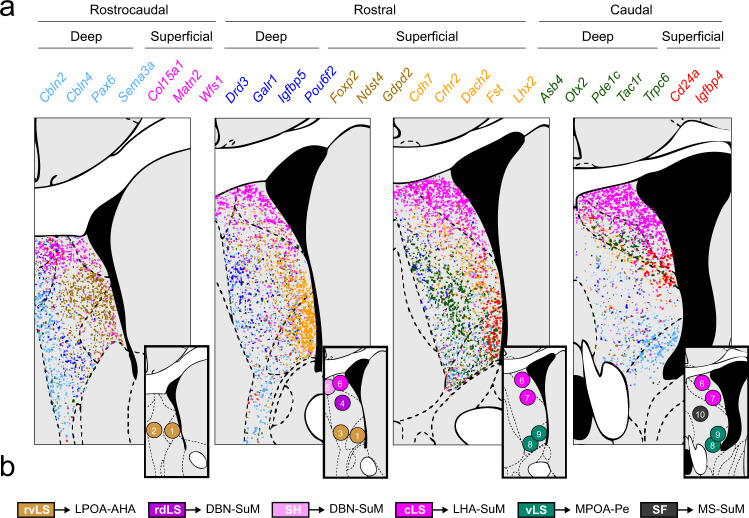
LS molecular profile and main outputs. **a** Gene expression domains defining non-overlapping LS territories. In situ hybridization data were annotated from mouse.brain-map.org and mapped at the level of four LS coronal planes (+1.1, +0.62, +0.38 and −0.1 mm from Bregma). **b** Principal LS territories defined by their descending projections. cLS: caudal, rdLS: rostrodorsal, rvLS: rostroventral, vLS: ventral, SF: septofimbrial nucleus, SH: septohippocampal nucleus. Summary of projection domains described by Risold and Swanson (1997b). 1: LS49, 2: LS36, 3: LS27, 4: LS17, 5: LS61, 6: LS40, 7: LS30, 8: LSRS, 9: LS53 and 10: LS50 (also called LS51). AHA: anterior hypothalamic area, DBN: diagonal band nucleus, LHA: lateral hypothalamic area, LPOA: lateral preoptic area, MPOA: medial preoptic area, MS: medial septum, Pe: periventricular nucleus, SuM: supramammillary nucleus.

The septum is a large central region of the brain that is separated from the striatum by the lateral ventricles. It is subdivided into medial, lateral, and triangular areas comprising different cell-types with different developmental origins and connectivity [1]. In rodents, the lateral septum (LS) is the largest septal region and shares some similarities to the striatum [2]. Both are mostly composed of GABAergic spiny neurons [3, 4] that receive topographically organized inputs from various cortical regions (Fig. 1a–f) [4, 5]. Rostral LS (rLS) receives dense glutamatergic inputs from the hippocampus, frontal (prefrontal cortex (PFC)) and temporal areas (entorhinal cortex, cortical amygdala and posterior amygdala). Along the rostro-caudal axis of LS, frontal and entorhinal inputs overlap near the midline, whereas amygdala inputs segregate near the ventricles. In contrast, the caudal LS (cLS) is almost exclusively innervated by the hippocampus. Ventral LS (vLS) receives prominent inputs from the amygdala and ventral hippocampus (vHPC). LS is organized like an onion with deep layers (toward the midline) and superficial layers (near the ventricles) generated at early and late stages of ontogeny, respectively [6, 7]. Although the idea of defining LS architecture based on selective gene expression domains is not new [8], open-source initiatives have allowed high-throughput brain-wide visualization of thousands of gene expression profiles [9]. Here, we provide for the first time a non-exhaustive list of discriminant markers that define deep and superficial gene expression domains along the rostrocaudal axis of LS (Fig. 2a). In addition, antidromic stimulation experiments suggest that all LS neurons send long-range projections to subcortical areas [10], particularly toward the hypothalamic and midbrain nuclei although their projections vary greatly along the dorsoventral, rostrocaudal and mediolateral axes (Fig. 2b) [8, 11]. Thus, cortical input territories intersect orthogonally with LS gene expression domains, leading to the poor correspondence between their respective topographical organization (Figs. 1–2). The mismatch between cortical projections and molecular organization suggests a complex transformation of cortical signals entering LS. Conversely, LS output territories overlap well with its gene expression domains (Fig. 2), thus suggesting a degree of correspondence between LS architecture and its long-range projections.

Based on the anatomy described above, LS is ideally positioned to integrate cortical signals in order to regulate the activity of hypothalamic and midbrain nuclei that control specific motivated behaviors. In contrast with the relatively well-characterized extrinsic connectivity of LS with cortical and subcortical brain regions, the intrinsic circuitry of LS remains largely unexplored, limiting our ability to understand how LS integrates and processes cortical signals. A major idea of this opinion piece is the importance of the largely unexplored intra-septal sub-circuits in LS function regulating motivated behaviors. We argue that the integration of cortical inputs enables the recruitment of different LS sub-circuits owing to its intrinsic connectivity. We hypothesize that these circuits are comprised of molecularly heterogeneous neurons with specific cortical inputs and sub-cortical outputs. We discuss the notion of parallel processing based on the existence of parallel circuits that process cortical signals independently, and of functional units that compete directly, each shaping the activity of one another. The main benefit of functional units over parallel pathways is the ability to orchestrate competing cortical signals that promote rival behaviors (e.g., food-seeking vs. aggression) in order to disinhibit a selective set of downstream subcortical nuclei and enable coordinated behavioral outcomes [12].

We first review classical lesion and stimulation studies that shed light on the critical role of LS in the regulation of motivated behaviors. We then explore how contemporary circuit-based studies of LS offer mechanistic insights into how it integrates cortical inputs to regulate hypothalamic and midbrain activity and associated motivated behaviors. Based on these key studies, we reject a global integration model and put forward parallel processing and functional unit models, contrasting them with the current state of the literature. This opinion piece aims at providing a blueprint for studying how LS molecular heterogeneity, intrinsic connectivity and output territories help to shape motivated behaviors.

## LS sub-circuits integrate heterogeneous cortical inputs to regulate distinct motivated behaviors

Early on, LS was proposed as a part of the limbic system for the regulation of mood, emotion and motivation [13, 14]. Pioneering studies in the 1950’s using male rats and cats showed that LS lesions elicit the so-called “rage syndrome” [15]. Around the same time, electrical stimulations applied to LS of rats were shown to exert potent rewarding properties enabling self-stimulation [16], a finding that paved the way for the characterization of the brain reward pathways. Many LS lesion and stimulation studies in rats ensued implicating LS in motivated behaviors that fulfill physiological needs such as water and food intake [17, 18], as well as safety needs such as the avoidance of threats and/or aggression (reviewed in [19]). LS lesions also impact adult social interactions [20–22], parental behaviors [23–25] and sexual behaviors [26]. This classic body of work suggests that LS contributes to the regulation of a wide variety of motivated behaviors [19, 27]. Because LS lesions often lead to exaggerated behavioral responses, LS was proposed to be part of a behavioral inhibition system exerting tonic inhibition onto various subcortical nuclei, and described as a “mood regulator” [19, 28].

Lesions applied to LS often yield opposing effects depending on their extent and location [19] and LS electrical stimulations were also shown to mediate bidirectional effects on the hypothalamus depending on which LS region is targeted [18]. These results raise the possibility that LS is heterogeneous in its action on the hypothalamus, and that intra-septal sub-circuits could interact with one another to select and relay optimal signals to downstream brain regions. Accordingly, recent work has shown that signals of opposing valence recruit non-overlapping yet intermingled neuronal populations within vLS [29].

**Fig. 3:**
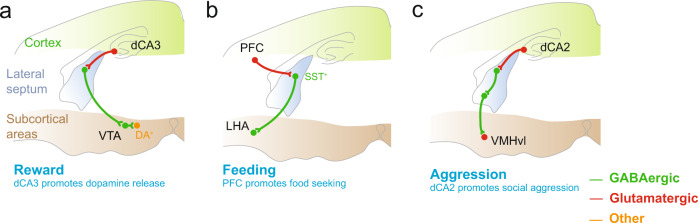
Illustrations of cortical circuits running through LS and their function. **a** Dorsal CA3 to VTA circuit regulating reward [30]. **b** PFC to LHA circuit promoting food-seeking behavior [32]. **c** Dorsal CA2 to VMHvl promoting social aggression [33]. LHA: lateral hypothalamic nucleus, PFC: prefrontal cortex, VMHvl: ventro-lateral part of the ventromedial hypothalamic nucleus, VTA: ventral tegmental area.

Recent projection-specific studies show that LS integrates discrete excitatory cortical inputs via intra-septal circuits. Indeed, an increasing number of studies have revisited the role of LS in motivated behaviors by using circuit-specific approaches. In a seminal study using rats, theta frequency stimulation of dorsal CA3 (dCA3) neurons was shown to inhibit ventral tegmental area (VTA) GABAergic neurons leading to the disinhibition of VTA dopaminergic neurons through a dCA3→dLS→VTA^GABA^→VTA^DA^ circuit (Fig. 3a) [30]. Lesions or chemogenetic inhibition within this circuit also prevented context but not cue-induced reinstatement of cocaine seeking [30, 31]. In mice, slow gamma oscillations (30–90 Hz) originating in the PFC are relayed through dLS neurons expressing somatostatin (dLS^SST^), which in turn project to the lateral hypothalamic area (LHA), causing an increase in the firing rate of a subset of LHA^GABA^ neurons in mice actively approaching food [32]. Optogenetic gain- and loss-of-function studies within this circuit revealed its critical role in food seeking but not consumption (Fig. 3b). We recently reported that dorsal CA2 (dCA2) of male mice promotes male aggression (but not predatory attacks) through activation of the ventro-lateral part of the ventromedial hypothalamic nucleus (VMHvl) [33]. Chemogenetic silencing was used to characterize a novel dCA2→dLS→rvLS→VMHvl disinhibitory circuit promoting male aggression (Fig. 3c). Specifically, dCA2 excites neurons in dLS that in turn inhibit neurons in vLS, which project to VMHvl [34, 35] causing its disinhibition and promoting male aggression [36]. Importantly, this recent study [33] went beyond previous work in highlighting the functional significance of intra-LS circuits. Overall, dLS neurons integrate different cortical excitatory inputs to facilitate motivated behaviors [30, 32, 33]. This is achieved through the recruitment of disynaptic disinhibitory circuits between dLS and vLS [33] or through an extra-septal inhibitory relay within downstream areas [30, 32]. In contrast to studies focusing on cortical projections to dLS, little is known on the role of direct cortical projections to vLS in regulating motivated behaviors, although recent studies using mice have begun to elucidate the role of specific rvLS populations in stress-induced adaptations [37, 38].

## Discounting a global integration of cortical inputs within LS

**Fig. 4:**
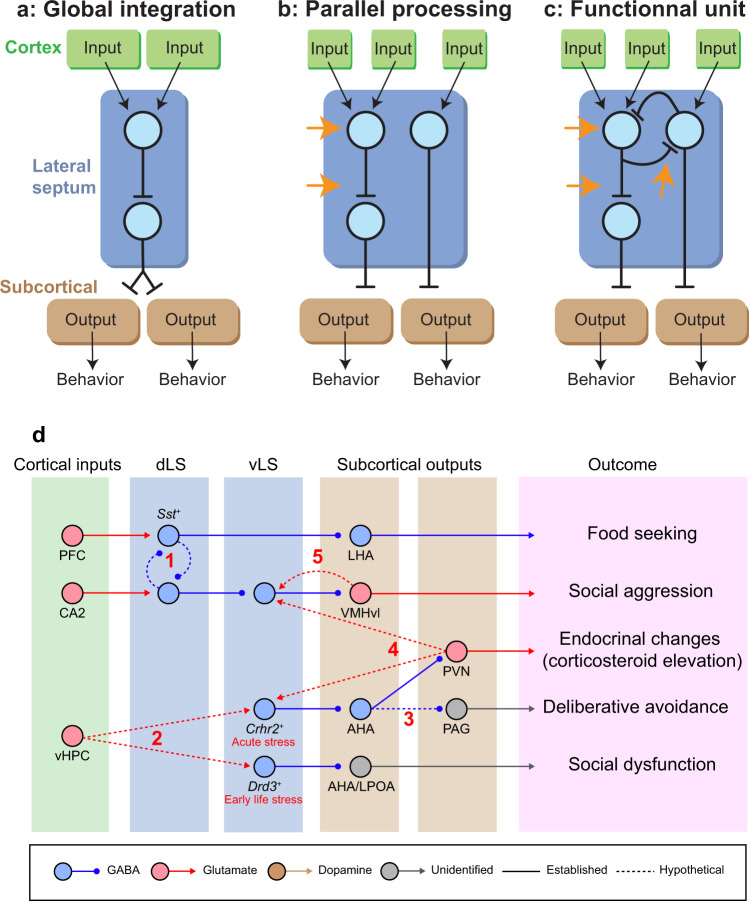
Functional models of LS circuits. **a** Cortical inputs are integrated together and LS broadcasts its inputs globally to provide the same level of inhibition onto all subcortical nuclei. **b** Distinct parallel circuits run alongside within LS to regulate different behaviors. **c** Parallel circuits with some degree of integration and lateral inhibition. Orange arrows represent the effect of neuromodulation at different levels of LS microcircuitry, differentially regulating each functional unit. We present three theoretical models of increasing complexity. The value of each model is discussed based on existing experimental evidence. **d** Examples of synergistic and antagonistic LS circuits organized around specific hippocampal inputs. Numbers in red represent distinct hypotheses.

The above-mentioned studies that focused on cortical-LS circuits raise as many questions as they answer. Are distinct cortical inputs to LS processed by separate parallel circuits or alternatively, do they converge onto common targets? If parallel circuits do exist, to what extent do they interact with one another? We consider three successive models of how LS processes cortical inputs in order to regulate motivated behaviors (Fig. 4). The first model (global integration model, Fig. 4a) surmises that cortical inputs are integrated together in LS before being broadcasted globally to provide homogenous disinhibition onto subcortical nuclei. In this framework, convergent cortical inputs would allow for regulating motivated behaviors all together. The global integration model is at odds with studies showing that the rat hippocampus projects topographically onto LS and that these projections are conserved all the way down to subcortical areas [5]. In addition, using cell-type specific retrograde trans-synaptic tracing tools in the mouse, we have recently demonstrated that dLS^SST^ neurons receiving monosynaptic inputs from the hippocampus receive very limited inputs from the PFC [39]. These results seriously undermine the global integration model and call for a better suited one.

## Heterogeneous integration of cortical inputs in LS sub-circuits

The second model implies the existence of distinct parallel circuits running alongside one another within LS to regulate different behaviors (parallel processing model, Fig. 4b), which could account for the heterogeneity of responses observed at the single-cell level [40]. Watts [41] proposed that the initiation of various behaviors requires the disinhibition of separate parallel circuits, including circuits running in and out of LS [2, 42], in order to yield different motor outcomes. In the parallel processing model, separate LS sub-circuits integrate and redistribute cortical information independently. One major limitation of the parallel processing model is the inability of LS to integrate synergistic cortical signals. Although LS cortical inputs are topographically organized, there is still a high degree of overlap among cortical input territories, especially in the rostral part (Fig. 1a) [11]. Thus, some degree of cortical input convergence onto parallel pathways appears likely and was included in the parallel processing model (Fig. 4b). The degree of convergence may, however, vary greatly between LS sub-circuits.

This second model predicts that parallel cortical inputs to LS recruit distinct subcortical regions to regulate different behaviors (Fig. 4b–c). This could occur through parallel LS circuits such as CA2-dLS disinhibiting VMHvl to promote aggression [33] and PFC-dLS disinhibiting a subset of LHA^GABA^ to promote food seeking [32]. Furthermore, different oscillation frequencies coupling within LS appear to differentially impact its downstream partners as coordinated slow gamma oscillations across the PFC→dLS→LHA circuit promote food seeking, unlike theta oscillations [32]. In contrast, coordinated theta oscillations across the dorsal HPC→dLS→LHA circuit are translated into rapid adjustments of running speed [43]. These observations suggest that different cortical circuits (e.g., PFC→dLS and HPC→dLS) may recruit LHA with distinct patterns of activity to promote different behaviors (i.e., food seeking vs. locomotor adjustment).

## Do LS inhibitory collaterals support the emergence of functional units?

Survival requires prioritizing and scaling multiple motivations concomitantly. This moment-to-moment selection of optimal behaviors should allow competing motivated behaviors such as food-seeking and aggression to occur in a mutually exclusive manner [12]. Here, we argue that lateral inhibition between LS intrinsic circuits shapes functional units that permit the selection of optimal behaviors (Model 3, Fig. 4c). Cortical signals coursing through LS and promoting competing behaviors (e.g., food seeking vs. aggression) would be ideally positioned to inhibit one another within LS, rather than in downstream areas. Experimental evidence collected in mice indicates that hunger decreases territorial aggression in the presence of food [44], raising the intriguing possibility that when food-seeking takes place, LS circuits supporting aggression may be actively suppressed. We therefore propose a third model (functional unit model, Fig. 4c), which consists of parallel circuits interacting through lateral inhibition mediated by abundant local collaterals. With lateral inhibition, LS intrinsic sub-circuits may enable the emergence of functional units as previously proposed in the striatum [45], competing to promote the most appropriate behavioral outcome. Importantly, we propose that functional units are not spatially segregated, but rather intermingled [29], as proposed in the striatum [45]. Functional units may thus be defined as an ensemble of neurons expressing heterogeneous molecular markers and projecting to several subcortical regions in order to regulate different aspects of a motivated behavior. We detail below the evidence arguing for the existence of functional units in LS.

Although most LS neurons send long-range projection to subcortical areas [10], several lines of evidence from rodent studies indicate that these neurons may also mediate lateral inhibition within LS. At the anatomical level, biocytin-filling of rat LS cells and injection of *Phaseolus vulgaris*-leucoagglutinin (PHAL) clearly show branching axons with long-range projections exiting ventrally together with collaterals that remain within LS [3, 46, 47]. Specifically, Staiger and Nurnberger observed *“[…] fibers originating from the different subnuclei of the lateral septal nucleus formed massive horizontal connections in the rostrocaudal axis. Projections to the contralateral, congruent subnuclei were also detected”* [46]. Consistently, anterograde trans-synaptic delivery of Cre recombinase in mice showed that cLS neurons innervated by dCA3 and dCA1 send long-range projections to the hypothalamus as well as collaterals to rLS [48]. These collaterals projecting to other LS regions are likely to provide lateral inhibition on antagonistic parallel pathways (see section *Possible interactions between LS circuits*). A similar lateral inhibition mechanism mediated by striatal medium-sized spiny neuron collaterals has been proposed to synchronize active ensembles and suppress unsynchronized cells in the striatum, allowing the emergence of functional units [45]. This observation is important, as the activity of distinct striatal ensembles promotes information transfer to downstream partners, thus defining the existence of functional units orchestrating specific behaviors [45]. In the same vein, collaterals arising from LS neurons can also impinge onto neurons from the same pathway to generate feedforward inhibition (FFI). Indeed, electrical stimulation of hippocampal afferents in rat LS slices elicits excitatory synaptic potentials followed by a late hyperpolarizing potential [49–51], a hallmark of FFI. In the cortex, FFI regulates the integration of incoming signals [52], while, in the hippocampus, FFI is known to facilitate neuronal firing following highly synchronous afferent excitatory postsynaptic potentials [53]. Potent FFI has been reported upon optogenetic stimulation of glutamatergic cortical [33, 54] and subcortical [55, 56] inputs to LS in mice. The role of FFI in LS remains to be determined but we propose that it could play an important role in the top-down coupling of oscillations between cortical and LS areas to regulate different behaviors [32, 43].

In summary, we propose that intra-septal collaterals mediate lateral inhibition in order to promote the emergence of functional units comprised of heterogeneous pools of LS neurons. In this framework, functional units integrate complementary inputs to facilitate a motivated behavior while suppressing antagonistic ones. Lateral inhibition may thus shape the activity of competing sub-circuits and orchestrate mutually exclusive behaviors.

## Possible interactions between LS circuits

We previously described two circuits running through LS and promoting food seeking or social aggression respectively [32, 33]. In these circuits, PFC pyramidal neurons projects to *Sst*^+^ neurons in rdLS to promote food seeking while CA2 pyramidal neurons projects to dLS neurons to facilitate social aggression (Fig. 4d1). Since food seeking and social aggression are mutually exclusive behaviors, we propose that lateral inhibition between rdLS and dLS allows one circuit to reinforce the inhibition onto the other, and therefore contributes to the selection of a unique behavioral outcome.

In contrast with studies focusing on cortical projections to dLS, little is known on the role of direct cortical projections to vLS. Recent studies in mice have nonetheless begun to elucidate the role of vHPC inputs to vLS in stress-induced responses. A recent report suggests that pyramidal neurons expressing *Coch* in vCA3 project onto vLS and play a role in acute social stress-induced deliberative avoidance [57]. Photo-silencing of vCA3 projections to LS however has no effect on deliberative avoidance in unstressed male mice [54]. Collectively, these results suggest that vCA3 projections to LS mediate deliberative avoidance in response to an acute stressor, specifically. Although the role of vHPC in deliberative avoidance has been the focus of numerous studies [58], whether vCA1 or ventral subiculum projections to LS control stress-induced deliberative avoidance remains to be established.

How could LS neurons broadcast vHPC signals to downstream subcortical brain regions in order to promote stress-induced responses? vHPC preferentially targets rvLS (Fig.1a–d), which can be segmented in superficial and deep layers based on a number of molecular markers, including *Crhr2* (superficial) and *Drd3* (deep, Fig. 2a). Consistently, cell-type specific monosynaptic rabies tracing indicates that *Drd3*-expressing (*Drd3*^+^) neurons receive prominent inputs from vHPC [38]. Future work should confirm whether *Crhr2*-expressing (*Crhr2*^+^) neurons also receive preferential inputs from vHPC and from which hippocampal region specifically (Fig. 4d2). Both superficial and deep layers in rvLS project to similar downstream partners such as LPOA and AHA (Fig. 2b). This is indeed the case for *Drd3*^+^ and *Crhr2*^+^ neurons which both target AHA [37, 38]. Interestingly, *Crhr2*^+^ neurons in rvLS mediate stress-induced persistent avoidance following acute stress while *Drd3*^+^ neurons are responsible for early life stress-induced social dysfunction. These two populations therefore display complementary roles in the physiological and behavioral manifestations of early life and acute stress.

*Crhr2*^+^ neurons inhibit GABAergic neurons in AHA, which disinhibits the paraventricular nucleus of the hypothalamus (PVN) and activates the hypothalamic–pituitary–adrenal axis in response to an acute stressor [37]. Silencing rvLS *Crhr2*^+^ neurons also impairs stress-induced avoidance behavior [37] leading Anthony et al. (2014) to propose that *Crhr2*^+^ neurons projecting to AHA also inhibit GABAergic neurons projecting to vlPAG to mediate stress-induced avoidance behavior (Fig. 4d3). In line with this hypothesis, a recent study found that AHA GABAergic neurons mediate avoidance behavior via their projection to the vlPAG [59]. Although few studies have focused on LS neuronal populations expressing specific molecular determinants [60], it is likely that discrete populations integrating information from similar cortical areas exert complementary yet non-overlapping roles on physiological and behavioral responses.

LS also receives abundant excitatory inputs from subcortical areas such as the PVN [56], LHA [61], VMHvl [62], SUM [63], and VTA [64]. These excitatory back-projections could very well participate in the regulation of functional units within LS. Recent work in the mouse demonstrated that PVN glutamatergic projections to rvLS potently prevent aggression while promoting avoidance behavior [56]. This interesting observation raises the intriguing possibility that acute stress could suppress aggression while promoting avoidance behavior by acting on rvLS neurons targeting VMHvl as well as *Crhr2* expressing neurons (Fig. 4d4).

Another exciting hypothesis is that subcortical glutamatergic projections to LS may act as negative feedback onto the very neurons that excite them in the first place in order to end a specific behavior. For instance, one could envision that glutamatergic VMHvl^ESR1^ neurons projecting to rvLS [62] could target the very neurons that inhibit them in the first place in order to terminate aggression (Fig. 4d5)? Similarly, LHA^GLUT^ neurons in mice potently suppress feeding [65] and send long-range projections to dLS [61]. Although the identity of dLS neurons targeted by LHA^GLUT^ remains elusive, a tantalizing hypothesis is that LHA^GLUT^ projections to dLS could interfere with PFC gamma entrainment in order to suppress food seeking [32]. Future work should aim at understanding how subcortical inputs to LS shape the integration of cortical signals in LS.

## Neuromodulation of LS sub-circuits to orchestrate the selection of motivated behaviors

One important feature of the rodent LS is that it receives extensive peptidergic inputs such as vasopressin (AVP), oxytocin [66–68], corticotropin-releasing factor (CRF) [8, 69, 70], enkephalin [71] or melanin-concentrating hormone [72] as well as cholinergic and monoaminergic inputs such as dopamine and serotonin [11, 73]. LS neurons themselves express neuromodulatory peptides such as enkephalin [5, 8]. These substances act through membrane receptors expressed by discrete LS neuronal populations which can be targeted with mouse genetics [37, 38]. Interestingly, these neuromodulatory receptors such as *Drd3*, *Galr1*, *Crhr2* and *Tac1r* are scattered across discrete gene expression domains within LS (Fig. 2a) and their effects on LS function remain mostly unknown except for a few studies [37, 38]. One attractive hypothesis is that the combination of neuromodulatory substances—reflecting the animal’s internal states and interoception [74]—may orchestrate the recruitment of specific intra-septal sub-circuits due to the heterogenous expression of their membrane receptors (Fig. 4, orange arrows). Future work should thus take into consideration the topographical organization of these neuromodulatory receptors in order to elucidate their impact on LS function.

To illustrate how neuromodulation may orchestrate the recruitment of intra-septal circuits, here we focus on the well-established action of AVP in LS to regulate rodent social behaviors (reviewed in [28, 75]). AVP inputs to LS originate from the medial amygdala and bed nucleus of the stria terminalis [76–78]. AVP injection in LS [79] of male rats enhances social discrimination and the deficit of social recognition exhibited by male mice lacking the AVP receptor 1a (V1a-KO mice) can be rescued using a viral vector injected into LS [80]. This suggests that AVP is released in LS of male rodents following an encounter with a novel conspecific to regulate social recognition [81]. Ex vivo studies in acute slices have proposed some potential mechanisms: AVP application on acute septal slices from rats induces long-term potentiation [82, 83] and application of a V1a agonist excites LS^V1a^ neurons which, in turn, inhibit LS neurons that do not express this receptor and are otherwise tonically active [84]—another example of disinhibition in LS. In order to fully understand the mechanism of action of AVP in LS, it will be crucial to investigate the inputs and outputs of LS neurons expressing AVP receptors in order to understand which intra-septal subcircuits are modulated. In addition, V1a-KO mice also exhibit changes in anxiety-related behaviors which can also be rescued using a viral vector strategy [80]. This suggests that V1a regulates different LS functions, perhaps through different circuits.

Septal release of AVP is also critical for male rodents during social aggression. Intra-LS injection of AVP promotes offensive aggression in rats [85] while V1b-KO mice exhibit decreased aggression [86]. We recently proposed a mechanism to reconcile these observations. Activation of presynaptic V1b on dCA2 pyramidal neuron terminals facilitates CA2 to dLS synaptic transmission and promotes aggression [33]. Altogether, these results suggest that, in male mice, V1a modulates LS circuits regulating social recognition and anxiety while V1b modulates LS circuits regulating aggression. Similar receptor-specific neuromodulation might also come into play with CRF receptors 1 and 2 expressed in distinct rodent LS territories [37, 87]. Little is known regarding the effect of other neuromodulatory substances released in LS, but testosterone implants in LS can rescue aggressive behavior in castrated male rodents [88] and dopamine promotes aggression in male mice [89]. This suggests that AVP is not the only modulator of LS circuits that regulates aggression. These results also raise the intriguing possibility that neuromodulation impacts LS function in a sex-specific manner given the important sex differences in neuromodulatory substances and receptors in this brain region [90, 91]. Interestingly, a recent study in female rats showed that AVP release in dLS prevented aggression, while oxytocin release in vLS promoted it [92], suggesting that LS neuromodulation regulates specific behaviors by changing the activity in defined regions. This also suggests that LS neuromodulation is sex-specific since AVP release in LS of male rodents has the opposite effect on aggression. In addition, LS output appears to be sexually dimorphic to some extent. For instance LS projections to the periaqueductal gray are denser in female rats, as shown with retrograde tracing tools [93]. Further investigation on the effect of each and of combined neuromodulatory substances onto LS sub-circuits activity is required. Finally, since most neuromodulatory inputs originate from the hypothalamus and midbrain, they may also act as a feedback mechanism in the LS.

## A roadmap to test the existence and functional significance of LS sub-circuits

Activity-dependent labeling strategies, trans-synaptic tracing approaches as well as high-throughput screening methods will be instrumental to test the predictions laid out in this perspective. Recent advances in activity-dependent labeling systems have catalyzed the study of the neural substrates for memory stabilization and recall [94]. The ability to target and study task-relevant neural ensembles within LS will make it possible to determine whether LS is organized as a collection of separate (model 2) or interacting sub-circuits (model 3). At the same time, it will be essential to capitalize on cell-type and projection-specific tools to evaluate whether LS parallel circuits act in a flexible and competitive manner to regulate a broad repertoire of motivated behaviors (models 2 and 3). Combining these approaches with longitudinal recordings and manipulations of LS sub-circuits will parse out their role in motivated behaviors and competing drive states. According to model 2, recruiting one sub-circuit should only facilitate one motivated behavior, while model 3 predicts that it might also suppress competing behaviors. For instance, live calcium imaging should capture the changes in activity preceding the initiation and completion of discrete behavioral motifs across LS parallel sub-circuits [39]. Optogenetic tools should also allow the direct recruitment of specific LS sub-circuits and therefore artificially bias the selection of motivated behaviors.

Mouse genetic systems combined with trans-synaptic tracing approaches allow tracing the relationship of inputs and outputs in highly divergent neural pathways (TRIO technique) [95]. Because LS sends long-range projections to a variety of downstream partners, the use of TRIO approach focused on LS itself or its downstream partners will be instrumental in refining our understanding of LS input-output connectivity. For instance, recent TRIO studies have demonstrated that the mouse LS projects onto the basal forebrain cholinergic neurons targeting PFC, but not amygdala, motor cortex or visual cortex [96]. Conversely, the mouse LS projects broadly onto pyramidal neurons located in ventral CA1 and ventral subiculum, irrespective of their downstream partners including PFC, NAc, and LHA [97, 98]. This is also true for mouse LS projections onto neurons in the supramammillary nucleus which in turn project onto DG or CA2 [99]. Although no studies have yet focused on LS exclusively, the use of the TRIO approach together, with mouse genetic systems will undoubtedly refine our understanding of LS input-output connectivity in a cell-type and projection-specific manner. This approach will be instrumental in identifying whether the convergence of cortical inputs in or downstream LS may regulate separately different aspects of motivated behaviors. For instance, a mouse PFC→dLS→LHA circuit promotes food seeking in a free-access feeding paradigm and a food-rewarded learning task [32]. In contrast, photo-silencing mouse vCA3 projections to dLS increases the latency when approaching food in a familiar but not novel environment [54]. Although PFC and vCA3 inputs to LS do not overlap within LS (Fig. 1), both ultimately converge toward LHA [11]. An attractive hypothesis is that frontal and hippocampal inputs to LS could impinge onto LHA to mediate different aspects of food seeking. TRIO approaches will enable targeting of frontal and hippocampal inputs to LS neurons projecting to the LHA and may resolve their differential contribution to food seeking. Applying this approach to other behaviors will disambiguate the selective contribution of convergent cortical inputs within or downstream of LS to the generation of complex behavioral responses depending on varying external stimuli and previous experiences.

Although all LS neurons send long-range projections to subcortical areas [10], it remains to be established whether single LS neurons project toward specific downstream partners or if they target multiple regions through axonal collateralization. Understanding to what extent LS neurons show axonal branching will be key to understanding LS function. If individual LS neurons show limited axonal branching, one can envision a division of labor amongst LS neurons mediating different processes of motivated behaviors. For instance, galanin-expressing neurons in the mouse medial preoptic area (MPOA^Gal^) mediate distinct sub-processes of parental behavior such as motor control, social interaction and motivational and neuroendocrine processes through projections to specific downstream regions [100]. In contrast, collateralization from single LS neurons could be biased toward distinct downstream partners, as reported in the mouse VMHvl [101]. In this regard, we have recently demonstrated that mouse dLS^SST^ neurons respond to electrical foot shocks, whose activity tracked and predicted non-freezing epochs in a context-specific manner [39]. These results clearly demonstrate that, unlike in the VMHvl, behavioral states can be mapped onto specific cell types in LS. Thus, cortical inputs could broadcast signals to complementary downstream brain regions to elicit different aspects of motivated behaviors, by either by recruiting distinct LS neurons (division of labor) or through LS neurons branching to project to multiple subcortical brain regions (collateralization). For instance, dopamine release is sustained during male mouse aggression [102] and VTA^DA^ stimulation is sufficient to promote male mouse aggression [103] at least in part through VTA^DA^ projections to LS [89]. Both dCA2 and dCA3 innervate overlapping territories in dLS [33] to promote aggression through the VMHvl or context-induced reinstatement of cocaine seeking through the VTA, respectively [30, 33]. An attractive hypothesis is that dCA2 also disinhibits VTA^DA^ neurons through a putative dCA2-dLS-VTA^GABA^-VTA^DA^ circuit. Future work should thus test whether single dLS or distinct dLS neurons disinhibit VMHvl and VTA^DA^ concomitantly to facilitate male rodent aggression. In this regard, the advent of high-throughput and projection-specific screening methods has allowed rethinking the classification of neurons into specific cell-types based on their projection features [104, 105]. This approach has successfully been employed to elucidate the highly divergent axonal projections of thousands of genetically barcoded neurons in mouse ventral CA1 [97]. Employing the same approach in LS will be instrumental in deciphering the complexity of LS parallel pathways with unprecedented granularity.

## Conclusion

We proposed three successive models for understanding how LS integrates and processes cortical inputs in order to regulate motivated behaviors. The first model of global integration can be rejected based on our knowledge of LS anatomy. The major feature of the last two models is the existence of distinct parallel pathways within LS, disinhibiting the activity of separate subcortical areas and therefore regulating different motivated behaviors. Some of these parallel circuits nonetheless integrate synergistic cortical signals and coordinate the activity of several subcortical nuclei respectively. The main difference between the second and third models lies in the critical role of lateral inhibition between parallel pathways. We propose that lateral inhibition within LS, akin to the inhibition described in the striatum [45] allows the formation of functional units that orchestrate motivated behaviors occurring in a mutually exclusive manner. It should be noted however that the LS and the striatum have different developmental origins [106], with different organization and connectivity [4, 11]. Thus, lateral inhibition could be a common mechanism across a broad range of GABAergic subcortical brain regions integrating cortical inputs such as LS, the striatum [45], and the central amygdala [107]. Recent work has emphasized that reciprocal inhibition between two populations could provide a ‘winner-take-all’ mechanism to generate a single behavioral outcome [108–110]. Although LS neurons display extensive local collaterals, which could support lateral inhibition, the existence of LS functional units remains to be established. This will require demonstrating that an ensemble of LS cells can facilitate a motivated behavior while preventing a rival one. In addition, functional units are thought to be highly dynamic [45], adjusting their activity depending on ever changing priorities. Although the current state of the literature does not allow us to make any predictions about which model, 2 or 3, may prevail, it is conceivable that while some LS sub-circuits interact with one another, others may act independently. Another prediction that arises from these models is that disruption of lateral inhibition could result in the inability to appropriately filter cortical inputs and alter the recruitment of functional units, leading to maladaptive behavioral responses. In this regard, early-life stress applied to mice was shown to reduce the ratio of excitatory to inhibitory inputs (E/I ratio) in a population of rvLS^DRD3^ neurons [38]. These effects were accompanied with long-lasting deficits in social behavior which could be normalized by restoring rvLS^DRD3^ activity [38]. Although the source of inhibitory inputs to rvLS^DRD3^ remains to be established, an intriguing hypothesis is that changes in the E/I ratio could stem from increased lateral inhibition proceeding from neighboring LS neurons.

In humans, very little attention has been paid to the septum verum, which is well developed when compared to other mammals [111]. Although the septum verum encapsulates the equivalent of the rodent lateral and medial septa [1], its overall organization remains poorly understood. The lack of understanding of its intrinsic connectivity precludes any speculation on the existence of parallel pathways, let alone functional units in the septum verum. In this regard, the development of probabilistic maps may facilitate the study of its structure and function [112, 113]. Nevertheless, developmental malformations or lesions of the septum verum have been associated with altered safety and social behaviors [1]. Alterations in safety and social processing are commonly observed in schizophrenia, autism spectrum disorder and bipolar disorder, which led Sheehan et al. (2004) to propose that LS dysregulation could be a central feature of these disorders. In addition, AVP modulation of LS is of high interest given the abnormal levels of AVP observed in patients with autism [114] and schizophrenia [115]. In addition, two case studies reported that damage to the septum verum led to excessive and inappropriate sexual behavior [116]. Characterizing the intrinsic connectivity and function of the septum verum is a major endeavor since dysregulation occurring within these circuits may lead to altered defensive and social processing [27, 117–119], a hallmark of many developmental and psychiatric disorders.
